# Mortality Risk Assessment in Peripheral Arterial Disease—The Burden of Cardiovascular Risk Factors over the Years: A Single Center’s Experience

**DOI:** 10.3390/diagnostics12102499

**Published:** 2022-10-15

**Authors:** Viviana Aursulesei Onofrei, Alexandr Ceasovschih, Dragos Traian Marius Marcu, Cristina Andreea Adam, Ovidiu Mitu, Florin Mitu

**Affiliations:** 1 “St. Spiridon” Clinical Emergency Hospital, Independence Boulevard nr 1, 700111 Iasi, Romania; 2 Department of Medical Specialties I, University of Medicine and Pharmacy “Grigore T. Popa”, University Street nr 16, 700115 Iaşi, Romania; 3 Clinical Rehabilitation Hospital, Cardiovascular Rehabilitation Clinic, Pantelimon Halipa Street nr 14, 700661 Iaşi, Romania

**Keywords:** peripheral artery disease, risk factors, prognosis, mortality risk, prognostic index, atherosclerosis

## Abstract

Atherosclerosis is the basis of the cardiovascular continuum in peripheral artery disease (PAD) patients. Limiting functional decline and increasing quality of life are the main objectives for these patients. We conducted a prospective cohort study on 101 patients with PAD admitted to a single center in Northeast Romania. We used an index score to evaluate the 10-year mortality risk assessment and based on the scores we divided the patients into two groups: a low and low-intermediate risk mortality group (49 cases, 48.5%) and a high-intermediate and high-risk mortality group (52 cases, 51.5%). We analyzed demographics, comorbidities, clinical and paraclinical parameters and we aimed to identify the parameters associated with an unfavorable prognosis. Patients in the high-intermediate and high-risk mortality group were associated more with cardiovascular risk factors. Hypertension (*p* = 0.046), dyslipidemia (*p* < 0.001), diabetes mellitus (*p* < 0.001), and tobacco use (*p* = 0.018) were statistically significant factors. Lipid profile (low-density lipoprotein cholesterol, *p* = 0.005) and fasting blood glucose (*p* = 0.013) had higher mean serum values in the high-intermediate and high-risk mortality group, with a positive correlation between them and the ankle-brachial index value (*p* = 0.003). A multidisciplinary assessment and, especially, correction of associated cardiovascular risk factors prevent complications, and thus, improve the prognosis in the medium and long term.

## 1. Introduction

Peripheral artery disease (PAD) is often accompanied by a multivessel disease leading to an increased risk of morbidity and mortality, and therefore, to a poor long-term prognosis in the absence of integrative, multidisciplinary management [1]. Endothelial dysfunction and hypercoagulable status induce the development of a thrombogenic and proinflammatory status at the level of the inferior vascular axis which determines the development and progression of both atherosclerotic lesions and their associated complications over time [2]. Diabetes mellitus, hypertension, dyslipidemia, and smoking are the main cardiovascular risk factors that accelerate the evolution of the pathophysiological processes mentioned above, thus, leading to increased prevalence and mortality in PAD [3].

The prevalence of PAD has doubled in recent decades as a result of accelerated industrialization, advances in technology, and increased life expectancy, which has led to both an increase in the incidence of obesity and diabetes and early diagnosis [4,5]. Worldwide epidemiological studies have shown that 5.6% of the global population suffer from PAD, with 90% of cases being asymptomatic or underdiagnosed [6]. PAD has been shown to be associated with a three-fold increased risk of mortality, with the existence of multivessel disease equaling or even exceeding the risk associated with patients with coronary artery disease (CAD) alone [5,7].

Patients with PAD have a very high association with cardiovascular risk of death or an acute vascular event. The clinical studies published in recent years have outlined the idea of different risk magnitudes of cardiovascular risk factors for the entities of polyvascular disease, but further pathophysiological studies are needed to understand the pathophysiological basis [3,8]. Thus, smoking, diabetes mellitus, hypertriglyceridemia, and microvascular damage induce a higher risk of PAD as compared with CAD or stroke, while serum low-density lipoprotein cholesterol levels predominantly influence the occurrence of CAD, and hypertension has a similar effect [2,9].

The lack of regional primary prevention programs contributes to maintaining a high prevalence of cardiovascular risk factors. In addition, despite the existence of good practice guidelines, patients with PAD are treated appropriately at a lower rate than those with the same risk factors and CAD [10,11,12]. Population ageing and continued digitization in the healthcare world urgently require the implementation of primary prevention programs to identify populations at risk. Early diagnosis of PAD could reduce the future medical and economic burden, and the application of integrative management could increase quality of life [6].

The aim of this study was to highlight the impact of traditional cardiovascular risk factors on the morbidity- and mortality-associated risk in PAD patients. Using the index score that assesses 10-year mortality risk, we investigated potential factors associated with a high risk of mortality using a cohort of PAD patients and, indirectly, the role of correcting these risk factors on preventing complications and improving long-term prognosis.

## 2. Materials and Methods

### 2.1. Study Design and PAD Diagnosis

We conducted a prospective cohort study on 101 patients diagnosed with PAD who had been admitted to a single tertiary center in Northeast Romania. The diagnosis of PAD was based on the presence of intermittent claudication (IC) associated with an abnormal ankle-brachial index (ABI) less than 0.9 or in the presence of symptoms suggestive of PAD and previously peripheral revascularization. IC has been defined as the occurrence of pain in the calf, thigh, or buttock, induced by exercise or ambulation and improved by rest [13]. In patients presenting with paresthesia, feeling of cold feet, pale and cold skin, reduced pilosity, subcutaneous atrophy, arterial ulcers, or dermatitis, ABI was performed to establish a positive diagnosis of PAD. The exclusion criteria were patients under the age of 18 years old and those with incomplete data regarding the parameters investigated. For patients with PAD enrolled in the study, demographics, personal medical history, tobacco and alcohol consumption habits, comorbidities, symptoms, clinical signs, paraclinical investigations, and chronic medication were obtained from the observation charts.

### 2.2. Measurements

**Figure 1:**
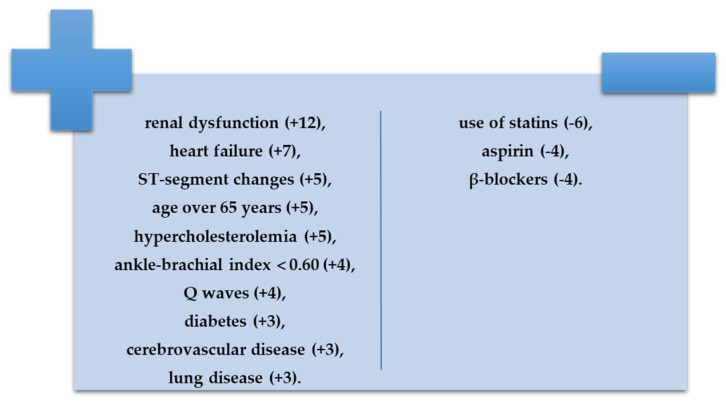
Risk index for 10-year mortality rate in PAD (adapted after [19]).

The most common associated comorbidities such as arterial hypertension [14], heart failure [15], chronic kidney disease [16,17], and diabetes mellitus [18] have been defined using current guidelines. The laboratory testing consisted of lipid profile (total cholesterol, low-density lipoprotein cholesterol, high-density lipoprotein cholesterol, tryglicerides), serum glucose, uric acid, renal function parameters (creatinine, urea), fibrinogen, and hemoleucogram. The results were presented according to the International System of Units. The body mass index (BMI) was calculated as the ratio of weight (kg) and height (m^2^). We used a validated prognostic index to indirectly assess the long-term mortality risk in PAD [19] and we calculated the scores of all patients at admission; on the basis of these scores, then, we divided the patients into two groups (a low and low-intermediate risk moortality group and a high-intermediate and high-risk mortality group). The risk index included various parameters presented below (Figure 1):

- With negative prognostic value, renal dysfunction (+12), heart failure (+7), ST-segment changes (+5), age over 65 years (+5), hypercholesterolemia (+5), ankle-brachial index less than 0. 60 (+4), Q waves (+4), diabetes (+3), cerebrovascular disease (+3), and lung disease (+3);
- With positive prognostic value, use of statins (−6), aspirin (−4), or β-blockers (−4).

Based on the scores, patients were divided into several risk categories with corresponding mortality rates: low (<0 points, 22.1%), low-intermediate (0–5 points, 32.2%), high-intermediate (6–9 points, 45.8%), and high (above 9 points, 70.4%) risk categories.

### 2.3. Statistical Analysis

Data were reported as the mean ± SD and as a number (frequency or percentages). The Kolmogorov–Smirnov test was used to assess the normal distribution of the data. Continuous variables were compared using the *t*-test (parametric analysis). Categorical variables were compared using the Fisher exact test. A *p*-value of ≤0.05 was considered to be statistically significant. To explore factors associated with clinical improvement at a 6-month follow-up, a regression analysis was performed. Receiver operating characteristic analyses were performed to calculate the area under the curve for clinical parameters. The descriptive analysis was performed using the SPSS statistics software (version 20 for Windows; SPSS Inc., Chicago, IL, USA).

### 2.4. Ethics

The study was approved by the Ethics Committee of the University of Medicine and Pharmacy, Grigore T. Popa ”Iași and of “St. Spiridon” Clinical Emergency Hospital and was conducted according to the Helsinki Declaration. All patients signed an informed consent statement which mentioned that the results would be used for research purposes.

## 3. Results

In our study, we enrolled 101 patients diagnosed with PAD who were evaluated and underwent integrative multidisciplinary management aimed mainly at correcting cardiovascular risk factors and improving functional decline. Patients were divided into the following two distinct subgroups according to the index score obtained, on the basis of which we indirectly assessed the risk of mortality 10 years after diagnosis: a low and low-intermediate risk mortality group (49 patients with a score of maximum 5 points) and a high-intermediate and high risk mortality group (52 patients with a score above 6 points).

Table 1 summarizes the statistical analysis of demographic data, vital parameters, predictors of index score, and cardiovascular risk factors or parameters derived from the clinical picture. In terms of demographics, we included patients previously diagnosed with PAD with a mean age of 70.67 ± 9.24, which were mainly male (51.5%). The percentage of patients over 65 years of age was higher in the high-intermediate and high-risk mortality group (63.3% vs. 73.1%, *p* = 0.692). Regarding anthropometric parameters, the average BMI at enrolment was 27.97 ± 4.88 kg/m^2^ with a higher mean value among high-intermediate and high-risk patients (27.55 ± 4.27 kg/m^2^ vs. 29.36 ± 5.40 kg/m^2^) and statistically significant value (*p* = 0.049).

Renal dysfunction (6.1% vs. 29.92%, *p* = 0.024), heart failure (18.4% vs. 32.69%, *p* = 0.019), hypercholesterolemia (51.0% vs. 65.4%, *p* < 0.001), and an ABI value below 0.6 (16.3% vs. 25.0%, *p* = 0.028) were comorbidities more frequently encountered by patients in the high-intermediate and high-risk mortality group, being also predictors with statistical value in assessing long-term prognosis. Among the main cardiovascular risk factors, smoking (32.7% vs. 51.92%, *p* = 0.018), obesity (38.8% vs. 59.61%, *p* = 0.041), and high blood pressure (61.2% vs. 90.4%, *p =* 0.046) were also more common in the high-intermediate and high-risk mortality group of patients. In terms of the presence of multivascular disease, CAD and cerebrovascular disease were more common in patients in the low and low-intermediate risk group.

The 101 PAD patients enrolled in the study presented in this paper were associated with a variety of clinical signs and symptoms. Thus, intermittent claudication (18.4% vs. 30.8%, *p* = 0.149), rest leg pain (22.4% vs. 25.0%, *p* = 0.763), and subcutaneous atrophy (8.2% vs. 13.5%, *p* = 0.393) were more frequently encountered in the high-intermediate and high-risk mortality group, while ulcers (4.1% vs. 1.9%, *p* = 0.523) were more frequent in the low and low-intermediate risk mortality group.

Table 2 contains biological data and paraclinical parameters, functional or associated with therapeutic management (drug agents or parameters associated with revascularization). Patients with a high risk of mortality at 10 years based on the index score were associated with higher mean serum values of total cholesterol (183.76 ± 47.20 mg/dL vs. 202.71 ± 46.68 mg/dL, *p* = 0.045), low-density lipoprotein (LDL) cholesterol (111.24 ± 41.18 mg/dL vs. 134.27 ± 40.93 mg/dL, *p* = 0.005), and triglycerides (140.41 ± 59.5 mg/dL vs. 150.27 ± 56.2 mg/dL, *p* = 0.058), as well as fasting glucose (114.18 ± 38.35 mg/dL vs. 130.65 ± 63.35 mg/dL, *p* = 0.013). Patients in the high-intermediate and high-risk mortality group were associated with a higher percentage of renal dysfunction, also evident in the case of serum creatinine (0.90 ± 0.26 mg/dL vs. 1.33 ± 0.41 mg/dL, *p* = 0.013) and urea (49 ± 19.19 mg/dL vs. 53.38 ± 25.33 mg/dL, *p* = 0.051), statistically significant parameters in our study. In addition, the high proportion of comorbidities in high-risk patients was reflected in the level of maximum walking distance, the average value in these patients being about 50% lower than in the low and low-intermediate risk patient group (352.86 ± 452.10 m vs. 148.33 ± 106.16 m, *p* = 0.145).

Patients enrolled in the present study benefited from integrative management, focusing on both drug treatment and revascularization of stenotic lesions or regular physical training. Thus, angiotensin-converting enzyme inhibitors (ACEIs) (44.9% vs. 48.1%, *p* = 0.385), angiotensin receptor blockers (ARBs) (4.1% vs. 9.6%, *p* = 0.314), or diuretics (34.7% vs. 53.8%, *p* = 0.822) were more frequently administered to patients in the second group, while beta-blockers (59.2% vs. 55.8%, *p* = 0.729) and aspirin (71.4% vs. 48.1%, *p* = 0.017) were more often recommended to patients in the first group (who more frequently associated CAD). Statins were administered to about 40% of patients in both groups (*p* = 0.965).

**Figure 2:**
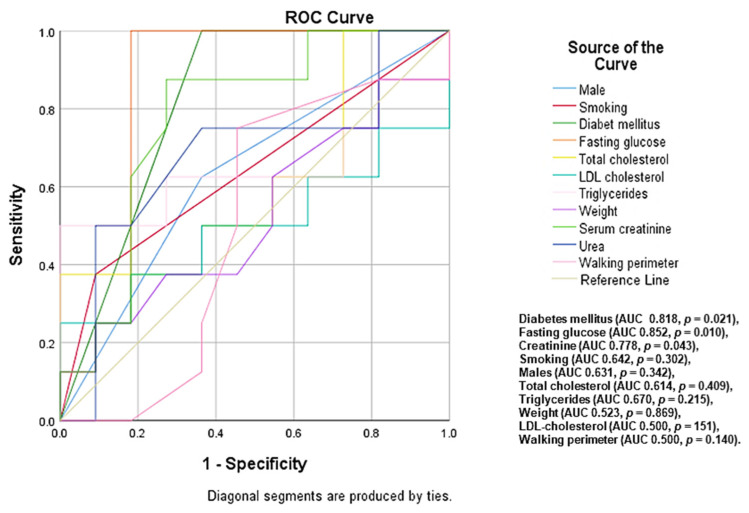
Predictors associated with a high risk mortality rate at 10 years.

Among the 101 patients with PAD, 32 (31.7%) patients had a high risk of 10-year mortality, with the predictors identified being diabetes mellitus (area under curve (AUC) 0.818, *p* = 0.021), high levels of fasting glucose (AUC 0.852, *p* = 0.010), serum creatinine (AUC 0.778, *p* = 0.043), smoking (AUC 0.642, *p* = 0.302), males (AUC 0.631, *p* = 0.342), total cholesterol (AUC 0.614, *p* = 0.409), triglycerides (AUC 0.670, *p* = 0.215), and weight (AUC 0.523, *p* = 0.869) (Figure 2).

Among the 101 patients with PAD, 24 (23.76%) patients were assessed angiographically, with two-sides disease (8.2% vs. 11.5%, *p* = 0.575, p* = 0.043), occlusive lesions (10.2% vs. 17.3%, *p* = 0.567), infrapopliteal location (37.5% vs. 45.5%, *p* = 0.080), and the presence of single atherosclerotic lesions (25.0% vs. 45.5%, *p* = 0.390) being more common in high-intermediate and high-risk patients. Patients in both groups underwent physical training, with a higher percentage of patients in the second group (53.1% vs. 61.5%, *p* = 0.389).

In a secondary level, the statistical analysis sought to identify predictors associated with a high risk of mortality at 10 years by comparing patients with the low and low-intermediate risk group and those only with high risk. In addition to clinical and paraclinical data with statistical value in the comparative analysis of the two groups, diabetes mellitus (*p* = 0.046), presence of erythema (*p* = 0.019), heart murmurs as an indirect marker of polyvascular disease (*p* = 0.006), serum levels of triglycerides (*p* = 0.046) and fibrinogen (*p* = 0.043), and two-sides disease (*p* = 0.043) are parameters associated with a high long-term risk in patients with PAD (Table 1 and Table 2).

## 4. Discussion

The occurrence and progression of atherosclerotic lesions is closely related to the presence of cardiovascular risk factors [20]. The lack of an integrative, multidisciplinary management of these patients, aimed both at improving the functional status and increasing the quality of life, leads in time to the appearance of complications and, implicitly, to negative prognoses. We used a validated prognostic index developed to identify long-term mortality risk in PAD and with its input, we identified a number of predictors with a prognostic role associated with a high risk mortality rate at 10 years.

In our study, the percentage of male PAD patients was higher (51.5% in the whole group), correlating with their predisposition to atherosclerotic lesions. Sex-related differences in PAD have been extensively studied in the literature and are strongly correlated with differences associated with arterial pathophysiological processes, hormones, menopausal onset, and serum estradiol levels [21,22,23]. Despite this, several clinical studies have been conducted on large cohorts of patients and have demonstrated a similar prevalence of PAD between the two genders [3].

In our study, the mean age was 70.67 ± 9.24 (*p* = 0.411), slightly higher in the group of patients with high-intermediate and high mortality risk (69.71 ± 10.01 vs. 71.58 ± 8.45), data similar to those presented by large clinical trials in the literature. Advancing age influences not only the prevalence of PAD, but also morbidity and mortality by accelerating functional decline and decreasing quality of life in the absence of treatment to address both the underlying pathology and the associated risk factors. Allison et al. [24] concluded that the prevalence of PAD doubled every decade, starting at age 40.

Smoking and diabetes mellitus are the main risk factors in PAD, which were also found in a high proportion in the clinical picture of the patients in our study. The percentage of smoking patients was higher among the second group (32.7% vs. 51.92%, *p* = 0.018), similar to the data presented in the literature. The harmful effect of smoking in patients with PAD has been demonstrated since 1911, when Erb et al. [25] pointed out that the risk of developing PAD was three times higher in smokers and six times higher in heavy smokers as compared with non-smokers. Smoking is a traditional cardiovascular risk factor that influences both therapeutic management and long-term patient prognosis. Thus, it has been shown that there was a more significant positive correlation between smoking and PAD as compared with CAD [26]. Patients who smoke develop PAD about 10 years earlier and have a two-fold risk of amputation [27]. Smoking cessation is essential, with data presented in the literature attesting to a dose-dependent relationship. Hooi et al. [28] demonstrated that active smokers had a 4.3-fold higher relative risk of developing PAD as compared with ex-smokers who were associated with a decreased risk of only 1.4. In a similar clinical study, another group of investigators demonstrated that the number of cigarettes also correlated directly with the risk of developing PAD; patients who smoked 1–14 cigarettes per day had a relative risk of 2.6, and those who smoked more than 25 cigarettes per day had a relative risk five times higher [29].

Diabetes mellitus tends to become the main risk factor for AOMI in the context of declining smoking prevalence in industrialized countries. In our study, 30.6% of patients with low and low-intermediate mortality risk and 40.6% of patients with high-intermediate and high mortality risk had diabetes mellitus (*p* = 0.051). Patients in the second group were also associated with elevated mean serum blood glucose values (114.18 ± 38.35 mg/dL vs. 130.65 ± 65.35 mg/dL, *p* = 0.013), which is a predictor with a negative impact on the risk of developing potential vascular complications. Prospective studies to date have shown that half of patients with diabetes will develop PAD within 30 years of diagnosis, with reduced glomerular filtration rate, female gender, and the need for concomitant insulin therapy and oral antidiabetic drugs being among the main associated risk factors [30,31].

The true prevalence of diabetes mellitus in PAD may be higher considering asymptomatic forms or the presence of peripheral polyneuropathy which alters pain perception [32]. Diabetic patients also have a higher risk of developing ischemic ulcers or gangrene as compared with non-diabetic patients with PAD [33]. The presence of these complications increases the risk of amputation up to 15-fold [34]. Insulin deficiency or altered tissue tolerance to circulating insulin accelerates the progression of lesions in the inferior vascular axis, the most significant changes being in the infrapopliteal arteries. In our study, two-sided disease (*p* = 0.043 for high mortality risk), presence of occlusive lesions (*p* = 0.567), or infrapopliteal artery damage (*p* = 0.080) were angiographic features more frequently encountered in intermediate-high and high-risk patients. These distally located lesions often present a therapeutic challenge in terms of severe calcification, long length, or reduced artery diameter [35].

Beckman et al. [36] demonstrated that diabetic patients with infrapopliteal atherosclerotic damage had reduced benefit from endovascular revascularization interventions. In a similar study, Favaretto et al. emphasized the negative impact of diabetes in patients with critical distal lesions, with prospective evaluation at 6 months indicating an increased rate of restenosis at the iliac or femuro-popliteal level after interventional revascularization of lesions at this level [37,38,39,40].

Patients in the second group had more comorbidities and cardiovascular risk factors as compared with those with a low and low-intermediate mortality risk at 10 years. In addition to smoking and diabetes mellitus, hypertension (*p* = 0.046), obesity (*p* = 0.041), dyslipidemia *p* < 0.001), renal dysfunction (*p* = 0.024), and heart failure (*p* = 0.019) were statistically significant parameters in our study. Hypertension was found in more than 50% of patients in both groups (61.2% vs. 90.4%, *p* = 0.046), representing, as in the clinical trials presented in the literature, a risk factor associated with both the development and progression of PAD. The negative impact on the risk of death occurs secondary to the increase in mean arterial pressure and vascular resistance which leads to increased arterial stiffness, and thus, to the development or progression of atherosclerotic lesions [41,42]. There is a causal relationship between systolic blood pressure (BP) and low ABI, with a 1.3 relative risk for hypertensive patients to develop PAD for every 10 mmHg increase in systolic BP [43,44]. In a similar clinical study, Lu et al. [45] found that patients with systolic BP values above 140 mmHg have a 2.6-fold higher associated risk of developing PAD. The optimal value of systolic blood pressure that, on the one hand, leads to lower mortality rate and, on the other hand, prevents the occurrence of ischemic events is a controversial topic, intensively studied in the literature. A recent study published by Sánchez Muñoz-Torrero et al. [46] concluded that, in patients with significant atherosclerotic disease (such as PAD or CAD), a decrease in SBP below 130 mmHg could lead to increased death rates.

Obesity has a significant impact on functional decline in patients with PAD, thus, interfering with independence, and thus, quality of life. In our study, obesity was present in 38.8% of the patients in the low and low-intermediate mortality risk group and in 59.61% of those in the high-intermediate and high-risk group (*p* = 0.041), representing, together with weight (*p* = 0.047) and BMI (*p* = 0.049), predictive factors for a high mortality rate at 10 years. It has been recognized that body mass index and abdominal circumference are the main parameters used to assess associated metabolic risk [47,48]. Heffron et al. [49] demonstrated that the strength of the statistical correlation between body mass index value and prevalence of PAD was influenced by BMI, with overweight patients (with a BMI of 25–29.9 kg/m^2^) having the lowest associated risk. The causal relationship between obesity and PAD has been extensively studied in the literature, with clinical studies showing controversial data [50]. Most studies mention the existence of a nonlinear relationship, in the form of the letter “J” [47]. Among the potential co-factors, gender played an important role; the association of the two entities was statistically more significant in women as compared with men [51].

Dyslipidemia contributes to the development of atherosclerotic lesions and their subsequent progression to unstable plaques responsible for potentially life-threatening acute vascular events [52]. In our study, hypercholesterolemia was objectified in 51.0% of the patients of the first group and in 65.4% of those of the second group, being a statistically significant parameter (*p* < 0.001) with a negative impact on the prognosis in PAD. In addition, patients at high risk of death at 10 years were associated with higher mean serum total cholesterol (*p* = 0.045), LDL-cholesterol (*p* = 0.005), and triglycerides (*p* = 0.058) values. Patients at high risk of death at 10 years were also associated with higher mean serum total cholesterol, LDL-cholesterol, and serum triglyceride values.

The prognostic value of lipid profile parameters has been demonstrated in clinical trials published in the literature to date. Murabito et al. [53] concluded in a prospective study of a significant cohort of 3313 patients that a serum total cholesterol level above 240 mg/dL or the association of lipid-lowering treatment was associated with a relative risk of 1.7 for identifying an ABI value below 0.9. Between serum triglyceride levels and progression of atherosclerotic lesions in patients with PAD, there was a statistically significant positive correlation (*p* < 0.01), with symptomatic patients associating a mean serum level 29 mg/dL higher on average as compared with patients without PAD [54]. A recent clinical study highlighted that non-high-density lipoprotein cholesterol, systolic BP, and smoking were the main risk factors responsible for the occurrence of acute vascular events in patients with symptomatic PAD [55]. In addition, the ratio of total cholesterol to HDL-cholesterol has been shown to be an independent prognostic factor associated with increased incidence of PAD among patients with type 2 diabetes [56].

Heart failure (*p* = 0.019), renal dysfunction (*p* = 0.024), and ABI values below 0.6 (*p* = 0.028) were more commonly present in patients with high 10-year mortality rates, thus, negatively influencing the prognosis of patients in the absence of integrated, multidisciplinary management. Heart failure and PAD frequently coexist in patients with atherosclerotic disease, the presence of the latter leading to increased risk of morbidity and mortality [57]. In a recent clinical study, Samsky et al. [58] identified age over 66 years, diabetes mellitus, and body weight under 76 kg as predictors of heart failure. The ABI value has also been correlated with the risk of developing HF; Nishimura et al. [59] and Gupta et al. [60] demonstrated, using statistical regression analysis, that an ABI value below 0.9 increased the risk of developing heart failure by 40% after adjusting for diabetes mellitus, smoking, CAD, or stroke. The prognosis of PAD patients with heart failure also depended on the type of heart failure (reduced ejection fraction vs. preserved ejection fraction) as well as on the associated treatment, considering the beneficial role of SGLT2 inhibitors on the prognosis of patients with heart failure regardless of the presence or absence of diabetes mellitus [61].

The prognostic value of ABI for assessing associated cardiovascular risk and mortality has been demonstrated in multiple clinical trials published to date. In our study, the ABI value was lower in patients at intermediate-high and high mortality risk, but without clinical significance (*p* = 0.519). The associated functional decline was clinically evidenced by measuring the maximum walking perimeter, the values obtained being significantly lower in patients from the intermediate-high and high-risk group (352.86 ± 452.10 m vs. 148.33 ± 106.16 m, *p* = 0.145), an aspect correlated with the higher number of associated comorbidities and the lower ABI value. Clinical studies in the field have attested to the predictive value of ABI at the level of the bilateral dorsalis pedis and posterior tibial arteries, with all-cause mortality rate (*p* < 0.001), cardiovascular mortality rate (*p* = 0.001), or the rate of occurrence of an acute cardiovascular event (*p* = 0.002) being directly proportional to the number of affected arteries [62].

A particular situation is represented by diabetic patients, in whom stiffened ankle arteries interfere with the correct assessment of ABI, requiring, instead, calculation of the toe-brachial index, taking into consideration the fact that stiffening of the toe arteries rarely occurs in diabetic patients [63,64].

ABI also correlates with serum creatinine; the presence of a low ABI value having predictive value for assessing the risk of death at 3 years in patients with chronic kidney disease [65]. In a similar clinical study, another group of researchers demonstrated that the association of a reduced glomerular filtration rate was associated with a high risk of mortality, independent of the presence of hypotension and diabetes mellitus, the value being additive to the other classic cardiovascular risk factors [66].

A significant percentage of PAD patients have simulated atherosclerotic disease in at least two arterial territories [4]. In our study, both cerebrovascular disease (*p* = 0.427) and CAD (*p* = 0.712) were more frequently encountered in low and low-intermediate risk patients. The more frequent concomitant coronary artery disease in patients of the first group justifies the presence of electrocardiographic changes (ST-segment changes, *p* = 0.094) predominantly in the first group of patients, as well as the increased percentage of patients on beta-blockers (*p* = 0.729) and aspirin (*p* = 0.017). Polyvascular disease is associated with a high risk of morbidity and mortality due to multiple associated comorbidities, polymedication, and lack of adherence to treatment, which over time negatively impacts prognosis by accelerating functional decline and decreases quality of life. Gutierrez et al. [67,68] demonstrated that the presence of multiple vascular bed involvement was associated with a higher risk of an acute cardiac event or increased need for revascularization of PAD lesions as compared with the presence of PAD alone.

Our study presents several limitations due to the number of cases analyzed and variability in the assessment of symptoms and clinical signs. We excluded those records where critical information was not available.

## 5. Conclusions

In our study, we demonstrated the role of identifying classical cardiovascular risk factors in patients with PAD, which have been shown to play a long-term prognostic role in increasing mortality. Diabetes mellitus, blood glucose, serum creatinine, smoking, lipid profile, male gender, and weight were the main predictors identified in our study to be associated with a high risk of death at 10 years among patients with PAD. Correcting them by adopting healthy lifestyles, combating sedentary lifestyles, and enrolling patients in extensive cardiovascular rehabilitation programs could combat the development of complications and could help to improve the risk of death estimated at 10 years.

## Figures and Tables

**Table 1:** Demographics, resting hemodynamics, and risk index parameters.

Paremeters	Total Group (*n* = 101)	Low and Low-Intermediate Risk Group (*n* = 49)	High-Intermediate and High-Risk Group (*n* = 52)	*p*-Value	p*-Value
**Demographics**					
Males	52 (51.5%)	30 (61.2%)	22 (42.3%)	0.270	0.289
Urban environment	38 (37.6%)	14 (28.6%)	24 (46.2%)	0.021	0.029
Age, y	70.67 ± 9.24	69.71 ± 10.01	71.58 ± 8.45	0.411	0.704
Height, m	1.65 ± 0.08	1.66 ± 0.07	1.64 ± 0.09	0.385	0.522
Weight, kg	76.53 ± 16.33	75.98 ± 13.95	79.06 ± 18.43	0.047	0.041
Body mass index, kg/m^2^	27.97 ± 4.88	27.55 ± 4.27	29.36 ± 5.40	0.049	0.031
**Resting hemodynamics**					
Systolic BP, mmHg	144.18 ± 19.44	142.29 ± 21.39	145.96 ± 17.43	0.345	0.754
Diastolic BP, mmHg	81.73 ± 7.40	80.71 ± 5.95	82.69 ± 8.49	0.181	0.557
Heart rate, bpm	82.55 ± 15.95	80.59 ± 13.05	84.40 ± 18.19	0.232	0.767
**Prognostic index risk factors**					
Renal dysfunction	17 (16.83%)	3 (6.1%)	14 (26.92%)	0.024	0.033
Heart failure	26 (25.74%)	9 (18.4%)	17 (32.69%)	0.019	0.038
Age > 65 years	69 (68.3%)	31 (63.3%)	38 (73.1%)	0.289	0.692
Hypercholesterolemia	59 (58.4%)	25 (51.0%)	34 (65.4%)	<0.001	0.031
ST-segment changes	37 (36.6%)	22 (44.9%)	15 (28.8%)	0.094	0.748
ABI < 0.6	21 (20.8%)	8 (16.3%)	13 (25.0%)	0.028	0.043
Q-waves	38 (37.6%)	16 (32.7%)	22 (42.3%)	0.317	0.387
Cerebrovascular disease	13 (12.9%)	7 (14.3%)	6 (11.5%)	0.427	0.574
Diabetes mellitus	38 (37.6%)	15 (30.6%)	23 (44.2%)	0.051	0.046
COPD	27 (26.7%)	12 (24.5%)	15 (28.8%)	0.621	0.830
**Other cardiovascular risk factors**					
Prior CAD or MI	24 (23.8%)	13 (26.5%)	11 (21.2%)	0.712	0.842
Smoking	43 (42.57%)	16 (32.7%)	27 (51.92%)	0.018	0.007
Obesity	50 (49.5%)	19 (38.8%)	31 (59.61%)	0.041	0.037
Hypertension	77 (76.2%)	30 (61.2%)	47 (90.4%)	0.046	0.021
**Symptoms and clinical signs**					
Intermittent claudication	25 (24.8%)	9 (18.4%)	16 (30.8%)	0.149	0.303
Rest leg pain	24 (23.8%)	11 (22.4%)	13 (25.0%)	0.763	0.842
Subcutaneous atrophy	11 (10.9%)	4 (8.2%)	7 (13.5%)	0.393	0.298
Erythema	7 (6.9%)	2 (4.1%)	5 (9.6%)	0.274	0.019
Ulcers	3 (3.0%)	2 (4.1%)	1 (1.9%)	0.523	0.231
Necrosis	2 (2.0%)	1 (2.0%)	1 (1.9%)	0.956	0.331
Heart murmur	40 (39.6%)	17 (34.7%)	23 (44.2%)	0.327	0.006
Right pedal pulse–absent	25 (24.8%)	14 (28.6%)	11 (21.2%)	0.388	0.341
Left pedal pulse–absent	22 (21.8%)	10 (20.4%)	12 (23.1%)	0.745	0.293
Right posterior tibial artery pulse–absent	15 (14.9%)	8 (16.3%)	7 (13.5%)	0.686	0.098
Left posterior tibial artery pulse–absent	15 (14.9%)	5 (10.2%)	10 (19.2%)	0.202	0.176
Arterial murmur	4 (4.0%)	2 (4.1%)	2 (3.8%)	0.952	0.769

All values are expressed as mean ± standard deviation (SD) or n (%); y, years; bpm, beats per minute; BP, blood pressure; ABI, ankle-brachial index; COPD, chronic obstructive pulmonary disease; CAD, coronary artery disease; MI, myocardial infarction. *p*—*p* value calculated for low and low-intermediate risk patient group vs. intermediate-high and high-risk patient group. p*—*p* value calculated for low and low-intermediate risk patient group vs. high-risk patient group.

**Table 2:** Risk factors included in the prognostic index.

Parameters	Total Group (*n* = 101)	Low and Low-Intermediate Risk Group (*n* = 49)	High-Intermediate and High Risk Group (*n* = 52)	*p*-Value	p*-Value
**Blood biochemistry**					
Total cholesterol, mg/dL	193.51 ± 47.65	183.76 ± 47.20	202.71 ± 46.68	0.045	0.020
LDL-cholesterol, mg/dL	117.95 ± 41.37	111.24 ± 41.18	134.27 ± 40.93	0.005	0.018
HDL-cholesterol, mg/dL	48.63 ± 12.52	46.77 ± 11.79	50.39 ± 13.04	0.147	0.293
Triglycerides, mg/dL	142.68 ± 101.02	140.41 ± 59.5	150.27 ± 56.2	0.058	0.046
Uric acid, mg/dL	5.94 ± 2.22	5.65 ± 1.96	6.25 ± 2.49	0.021	0.044
Creatinine, mg/dL	0.95 ± 0.35	0.90 ± 0.26	1.33 ± 0.41	0.047	0.038
Fasting glucose, mg/dL	122.66 ± 54.33	114.18 ± 38.35	130.65 ± 65.35	0.013	0.028
Urea, mg/dL	48.68 ± 22.45	49 ± 19.19	53.38 ± 25.33	0.051	0.059
Fibrinogen	385.33 ± 83.41	368.92 ± 67.39	400.79 ± 94.15	0.055	0.043
Hematocrit	40.83 ± 5.78	41.42 ± 5.95	40.27 ± 5.62	0.320	0.114
Platelets	242,554.46 ± 77,639.74	247,795.92 ± 88,404.84	237,615.38 ± 66,426.77	0.513	0.519
**ABI**	0.87 ± 0.31	0.89 ± 0.29	0.85 ± 0.32	0.519	0.269
**Walking perimeter (m)**	223.68 ± 292.05	352.86 ± 452.10	148.33 ± 106.16	0.145	0.371
**Right toe SBP**	94.62 ± 33.23	91.63 ± 32.44	97.44 ± 34.04	0.383	0.687
**Left toe SBP**	94.34 ± 28.69	99.23 ± 28.15	89.83 ± 28.71	0.102	0.265
**Left toe-brachial index**	0.70 ± 0.23	0.67 ± 0.23	0.72 ± 0.22	0.301	0.452
**Right toe-brachial index**	0.70 ± 0.22	0.73 ± 0.19	0.67 ± 0.23	0.230	0.656
**LV ejection fraction (%)**	54.77 ± 13.78	56.98 ± 12.28	52.43 ± 14.98	0.097	0.898
**Medication**					
ACE inhibitors	47 (6.5%)	22 (44.9%)	25 (48.1%)	0.385	0.749
Angiotensin receptor blockers	7 (6.9%)	2 (4.1%)	5 (9.6%)	0.314	0.475
Diuretics	45 (44.6%)	17 (34.7%)	28 (53.8%)	0.822	0.601
Calcium blockers	25 (24.8%)	12 (24.5%)	13 (25.0%)	0.407	0.624
Beta-blockers	46 (45.5%)	29 (59.2%)	29 (55.8%)	0.729	0.304
Alpha blockers	3 (3.0%)	2 (4.1%)	1 (1.9%)	0.279	0.769
Aspirin	60 (59.4%)	35 (71.4%)	25 (48.1%)	0.017	0.190
Clopidogrel	8 (7.9%)	3 (6.1%)	5 (9.6%)	0.672	0.724
Warfarin	11 (10.9%)	3 (6.1%)	8 (15.4%)	0.648	0.374
Trifusal	4 (40%)	3 (6.1%)	1 (1.9%)	0.003	0.088
Statins	41 (40.6%)	20 (40.8%)	21 (40.4%)	0.965	0.666
Antidiabetic medication	20 (19.8%)	8 (16.3%)	12 (23.1%)	0.395	0.722
Insulin	8 (7.9%)	2 (4.1%)	6 (11.5%)	0.165	0.246
**Angiography**					
Collateral circulation	16 (15.8%)	8 (16.3%)	8 (15.4%)	0.557	0.817
Two-sides disease	10 (9.9%)	4 (8.2%)	6 (11.5%)	0.575	0.043
**Severity of lesions**					
Stenosis	8 (7.9%)	6 (12.2%)	2 (3.8%)	0.567	0.791
Complete occlusion	14 (13.9%)	5 (10.2%)	9 (17.3%)		
**Location of lesion**					
Suprapopliteal	16 (84.2%)	7 (87.5%)	9 (81.8%)	0.754	0.947
Infrapopliteal	8 (42.1%)	3 (37.5%)	5 (45.5%)	0.080	0.157
**Number of lesions**					
Single	7 (6.8%)	2 (25.0%)	5 (45.5%)	0.390	0.238
Multiple	12 (63.2%)	6 (75.0%)	6 (54.5%)		
**Invasive treatment**					
Interventional revascularization	5 (5.0%)	3 (6.1%)	2 (3.8%)	0.598	0.682
Surgical revascularization	13 (12.9%)	5 (10.2%)	8 (15.4%)	0.437	0.940
**Exercise training**	58 (57.4%)	26 (53.1%)	32 (61.5%)	0.389	0.871

All values are expressed as *n* (%) or as mean ± standard deviation (SD). LDL-cholesterol, low-density lipoprotein cholesterol, HDL-cholesterol, high-density lipoprotein cholesterol; SBP, systolic blood pressure; LV, left ventricle; ACE, angiotensin-converting enzyme. *p*—*p* value calculated for low and low-intermediate risk patient group vs. intermediate-high and high-risk patient group. p*—*p* value calculated for low and low-intermediate risk patient group vs. high-risk patient group.
